# Associations between KIR/KIR-ligand genotypes and clinical outcome for patients with advanced solid tumors receiving BEMPEG plus nivolumab combination therapy in the PIVOT-02 trial

**DOI:** 10.1007/s00262-023-03383-w

**Published:** 2023-02-23

**Authors:** A. S. Feils, A. K. Erbe, J. Birstler, K. Kim, U. Hoch, S. L. Currie, T. Nguyen, D. Yu, A. O. Siefker-Radtke, N. Tannir, S. M. Tolaney, A. Diab, P. M. Sondel

**Affiliations:** 1 Department of Human Oncology, University of Wisconsin School of Medicine and Public Health, Madison, WI USA; 2 Department of Biostatistics and Medical Informatics, University of Wisconsin School of Medicine and Public Health, Madison, WI USA; 3 University of Wisconsin Carbone Cancer Center, Madison, WI USA; 4 Nektar Therapeutics, San Francisco, CA USA; 5 Virion Therapeutics, Newark, DE USA; 6 University of Texas MD Anderson Cancer Center, Houston, TX USA; 7 Department of Medical Oncology, Dana-Farber Cancer Institute, Boston, MA USA; 8 Department of Pediatrics, University of Wisconsin School of Medicine and Public Health, Madison, WI USA

**Keywords:** Nivolumab, Bempegaldesleukin, Killer immunoglobulin-like receptor (KIR), NK cells, Human leukocyte antigen (HLA)

## Abstract

Bempegaldesleukin (BEMPEG), a CD122-preferential IL2 pathway agonist, has been shown to induce proliferation and activation of NK cells. NK activation is dependent on the balance of inhibitory and excitatory signals transmitted by NK receptors, including Fc-gamma receptors (FCγRs) and killer immunoglobulin-like receptors (KIRs) along with their KIR-ligands. The repertoire of KIRs/KIR-ligands an individual inherits and the single-nucleotide polymorphisms (SNPs) of FCγRs can influence NK function and affect responses to immunotherapies. In this retrospective analysis of the single-arm PIVOT-02 trial, 200 patients with advanced solid tumors were genotyped for KIR/KIR-ligand gene status and FCγR SNP status and evaluated for associations with clinical outcome. Patients with inhibitory KIR2DL2 and its ligand (HLA-C1) observed significantly greater tumor shrinkage (TS, median change −13.0 vs. 0%) and increased PFS (5.5 vs. 3.3 months) and a trend toward improved OR (31.2 vs. 19.5%) compared to patients with the complementary genotype. Furthermore, patients with KIR2DL2 and its ligand together with inhibitory KIR3DL1 and its ligand (HLA-Bw4) had improved OR (36.5 vs. 19.6%), greater TS (median change −16.1 vs. 0%), and a trend toward prolonged PFS (8.4 vs. 3.6 months) as compared to patients with the complementary genotype. FCγR polymorphisms did not influence OR/PFS/TS.

These data show that clinical response to BEMPEG plus nivolumab treatment in the PIVOT-02 trial may be associated with the repertoire of KIR/KIR-ligands an individual inherits. Further investigation and validation of these results may enable KIR/KIR-ligand genotyping to be utilized prospectively for identifying patients likely to benefit from certain cancer immunotherapy regimens.

**Supplementary Information:**

The online version contains supplementary material available at 10.1007/s00262-023-03383-w.

## Background

Within the past decade, significant advances in immunotherapy have led it to be an effective treatment option for a range of solid tumors [1–5]. Immunotherapeutic regimens comprised of immune checkpoint inhibitors (ICIs), such as anti-programmed death-1 (anti-PD-1) and high dose IL2 (HD-IL2) are among those that have shown clinical benefit, however, only in a select subset of patients [6, 7]. Thus, improved therapeutic agents and combination regimens are necessary to provide clinical benefit for a larger population of patients.

The PIVOT-02 Phase I/II clinical trial (NCT02983045) enrolled patients with advanced solid tumors to evaluate the efficacy of a combination therapy consisting of nivolumab (anti-PD-1) and a novel agent, bempegaldesleukin (BEMPEG). As a CD122-preferential IL2 pathway agonist, BEMPEG serves to address some of the limitations seen by ICIs and HD-IL2. Compared to HD-IL2, BEMPEG preferentially binds to the low-to-moderate affinity heterodimeric IL2βγ (CD122/132) receptors predominately expressed on NK and CD8 T cells, compared to the high-affinity trimeric IL2αR predominately expressed on immunosuppressive T regulatory cells (Tregs), and stimulates an anti-tumor immune response through the clonal expansion of NK and CD4 and CD8 T cells. A Phase I trial for BEMPEG monotherapy confirmed its ability to induce proliferation and activation of T cells and NK cells in the blood and tumor microenvironment (TME) [8]. Thus, in this retrospective analysis of data from the completed PIVOT-02 trial, considering BEMPEG can activate patients’ NK cells, we sought to investigate whether distinct immunogenotypes related to NK cell function were associated with patients’ clinical outcome from the combination therapy of BEMPEG plus nivolumab.

NK cell activation is dependent on the balance of inhibitory and excitatory signals transmitted by receptors expressed on NK cells, including killer immunoglobulin-like receptors (KIRs) and Fc-gamma receptors (FCγRs). Different KIRs can have an inhibitory or excitatory function as they interact with their corresponding HLA molecules (KIR-ligands) expressed on healthy or cancerous cells. FCγRs are expressed on NK cells, as well as other immune cells, and can influence NK cell function through binding to the fragment crystallizable (Fc) region of tumor-bound antibodies, subsequently leading to anti-tumor responses through triggering of antibody-dependent cellular cytotoxicity (ADCC). We, and others, have shown that the repertoire of KIRs and KIR-ligands an individual inherits and the single-nucleotide polymorphisms (SNPs) among FCγRs can influence NK cell function and affect responses to certain immunotherapies [9–14]. Here, we focus on the association between clinical response and the presence or absence of the four inhibitory KIRs and their ligands that we have previously found associated with clinical outcome [9–11]: KIR2DL1 with HLA-C2; KIR2DL2 and KIR2DL3 with HLA-C1; and KIR3DL1 with HLA-Bw4 epitopes. We acknowledge that the KIR2DL2 inhibitory receptor is in linkage disequilibrium with the KIR2DS2 activating receptor, and as such, some of our findings might reflect the influence of activating receptors, as we previously detailed [9]. We also report on our findings looking for potential associations of FCγRs, alone and in combinations, with clinical outcome. In this report, we found that there were no associations with FCγR combinations and clinical outcome, but similar to our prior observations with KIR/KIR-ligand genotypes, we found that certain combinations of KIR/KIR-ligand genotypes associate with clinical outcome.

## Methods

### Clinical trial and clinical samples

PIVOT-02, a phase I/II dose-escalation/expansion trial (NCT02983045), evaluated the safety and efficacy of BEMPEG in combination with nivolumab in selected advanced or metastatic solid tumors. All patients included in our analyses were treated with the recommended phase 2 dose (RP2D) of 0.006 mg/kg BEMPEG every 3 weeks plus 360 mg nivolumab every 3 weeks until disease progression, death, unacceptable toxicity, symptomatic deterioration, investigator decision to discontinue treatment, patient withdrawal of consent, loss to follow-up, or study termination by the sponsor. Responding patients were treated for a maximum of 2 years. For this retrospective analysis, we focused on a subset of patients from the PIVOT-02 trial who had not previously been treated with immunotherapy (IO-naïve). We excluded 13 patients previously treated with immunotherapy for whom we analyzed DNA, in order to have a more homogeneous population of 200 IO-naïve patients for our analysis. As these 13 patients previously treated with immunotherapy would be expected to have worse outcome than the IO-naïve patients, the random distribution of these few patients into the various genotyping groups evaluated here could influence the associations of clinical outcome with genotype. Supplementary Table S1 includes the patients who received prior immunotherapy (RP2D cohort, *n* = 213) and displays the results for the four associations reported on in this manuscript. A breakdown of the IO-naive patient subset according to tumor type is shown in Table 1. The clinical details of the PIVOT-02 trial and its phase I/II clinical conclusions have been reported by Diab et al. [15–18]. The study was conducted in accordance with Good Clinical Practice guidelines and the Declaration of Helsinki. All patients provided written informed consent, and the protocol was approved by independent ethics committees and the institutional review board at each participating site.

**Table 1:** PIVOT-02 tumor cohort enrollment. Patients with selected advanced solid tumors were enrolled in the PIVOT-02 Phase I/II trial. All patients analyzed in this retrospective study received the RP2D. All results reported here, unless otherwise specified, are of the IO-naïve cohort (*n* = 200). 1L = first-line therapy; 2L = second-line therapy

Tumor Type	*n*
1L melanoma (MEL)	31
Advanced melanoma progressing after adjuvant therapy	7
1L renal cell carcinoma (RCC)	37
Renal cell carcinoma (other)	3
1L Metastatic urothelial cancer (mUC)	37
1-2L Triple-negative breast cancer (TNBC)	33
1-2L non-small cell lung cancer (NSCLC)	49
Non-small cell lung cancer (other)	3
**Immunotherapy (IO) Naïve**	**200**

The bold in Table 1 is highlighting the sum of all patients across all tumor types (n=200) being immunotherapy-naive

### DNA isolation and whole-genome amplification

A total of 200 IO-naïve patients from the PIVOT-02 trial had DNA available for genotyping, along with clinical data for correlative analyses. Genomic DNA was isolated from peripheral blood mononuclear cells following the manufacturer’s protocol of the DNeasy Blood and Tissue Kit (Qiagen). If necessary, DNA was then whole-genome amplified using the REPLI-g Mini Kit as per the manufacturer’s protocol (Qiagen) and used for KIR/KIR-ligand and FCγR genotyping.

### Genotyping

Frequencies for KIR/KIR-ligand and FCγR genotypes, shown in Supplementary Table S2, are similar to those reported by others for these genes [13, 19–26]. For simplicity, abbreviations for the specific genotype groups are used throughout the manuscript. The distinct genotypes comprising all the possible combinations assessed in these analyses are detailed in Supplementary Tables S3 and S4.

#### KIR/KIR-ligand

KIR gene status was assessed by real-time SYBR green PCR melt curve analyses as developed by Vilches et al. [27]. The KIR-ligand genotypes (HLA-C1, HLA-C2, HLA-Bw4) were determined by sequence specific primers-polymerase chain reaction (SSP-PCR) using the KIR HLA Ligand SSP Typing kit (Olerup) with GoTaq DNA Polymerase (Promega).

#### Fc-gamma receptor

FCγR SNP status for FCγR2A was determined using Taqman primers/probes. For FCγR3A and FCγR2C SNP status, RNaseH primers/probes were used with Taqman Fast Advanced master mix (ThermoFisher) [28]. For FCγR2C, a modified protocol was used to test for FCγR2C SNP rs759550223 as follows: A total reaction volume of 5uL included Taqman Fast Advanced master mix (ThermoFisher), 0.5uM each of the forward (5′-TCTCCCTCTCTCTTTATCCTTCTG-3′) and reverse (5′-TGTCAGAGTCACAGAGTCCTC**rU**TGGAC- C3spacer-3′) primers, both in TE buffer (pH 7.5); 0.5uM each of the FCγR2C-C (5′-ATTO532N CAC+T+G+GGG+CT-3′ _Iowa Black FQ Quencher) and FCγR2C-T (5′-FAM TCCAC+T+A+GGG+CT-3′ _Iowa Black FQ Quencher) probes, both in TE buffer (pH 8.0); 5 mU of RNase H2 Enzyme (IDT DNA); and 2.5 ng of DNA. The thermocycler conditions included a pre-read at 60C for 30 s, a 95C hold for 20 s, followed by 50 cycles of 95C for 1 s with 63C for 20 s, and a post-read at 60C for 30 s. FCγR2C SNP calls were made based on the amplification curves.

### Data management

All KIR/KIR-ligand and FCγR genotyping was conducted in a blinded manner, whereby individuals who determined the genotype of the patients did not have access to the clinical outcome data. Genotyping results were collected and managed using REDCap electronic data capture tools hosted at the University of Wisconsin-Madison [29, 30]. The clinical outcome data from the PIVOT-02 study database were merged with the genotyping data in REDCap to create a SAS dataset for analysis.

### Statistical analyses

Genotyping results were analyzed for association with the following clinical outcome parameters: (1) objective response (OR); (2) progression-free survival (PFS); and (3) maximum percentage tumor shrinkage (TS, defined as the best percentage change in tumor size from baseline). All 200 patients were included in the OR and PFS analysis. Three patients were excluded from TS analysis, as they did not have measurable disease per RECIST v1.1 at baseline and ≥ 1 post-baseline tumor response assessment. Tumor shrinkage as an additional parameter for clinical outcome has been utilized in our prior publications of associations between immunogenotypes and clinical outcome along with published clinical reports of this PIVOT-02 trial [10, 11, 17, 18]. The confirmed best overall response (CBOR) was evaluated by RECIST v1.1; PD, progressive disease; SD, stable disease; PR, partial response; CR, complete response. The OR rate (ORR) was defined as the percentage of the 200 patients with a CR or PR. The Kaplan–Meier method was used for estimation of the survival distribution for PFS. Comparative analyses were evaluated using a binomial test for OR, log-rank test for PFS, and Wilcoxon rank-sum test for tumor shrinkage. All tests conducted were two-sided. Analyses resulting in *p* values less than 0.05 were considered significant. Due to the relatively small patient population in this study, analyses resulting in *p* values less than 0.1 were considered trends. In the waterfall plots, dotted red boxes outline the data for the top third percent of patients with a positive clinical response (based on OR and tumor shrinkage parameters), to provide a visual “reference standard” to enable easier visual comparisons of the clinical benefit seen from each genotype at an individual patient level.

## Results

### KIR-ligand present/missing status does not significantly influence clinical outcome in IO-naïve patients

**Fig. 1:**
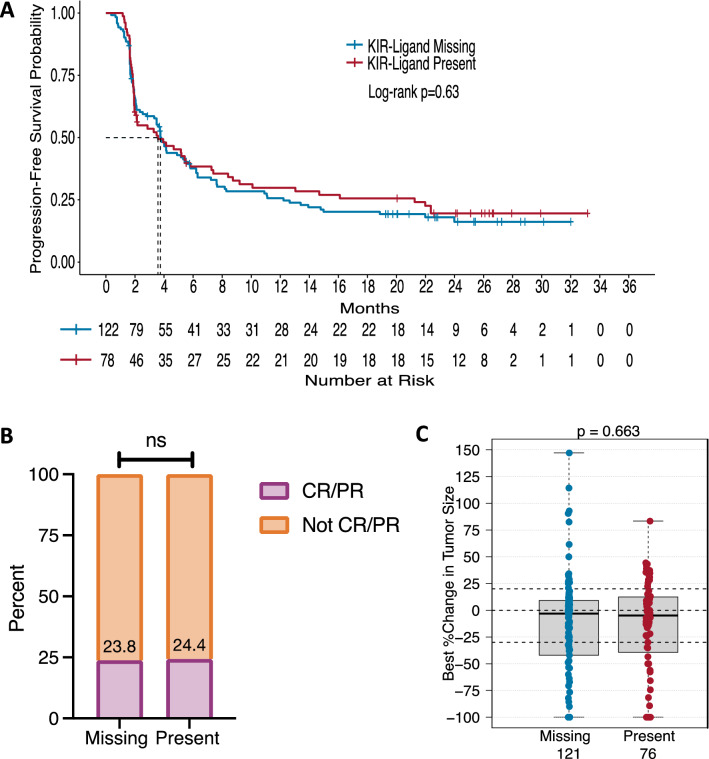
KIR/KIR-ligand present/missing status does not influence clinical response to BEMPEG plus nivolumab therapy for IO-naïve patients. Kaplan–Meier curve for PFS **a** compares patients with a KIR-ligand missing genotype (blue line) to patients with a KIR-ligand present genotype (red line). OR is represented in (**b**) by the proportion of patients with a CR/PR (purple) compared to patients without a CR/PR (orange) based upon their KIR/KIR-ligand present/missing status. The box plots in (**c**) compare tumor shrinkage as indicated by the percent change in target lesion size from baseline for patients with a KIR-ligand missing genotype (blue) to patients with a KIR-ligand present genotype (red). Dotted horizontal lines indicate a +20% increase and −30% decrease in target lesion size from baseline

Mature NK cells that express inhibitory KIRs mediate reduced tumor-targeted direct killing or ADCC when the KIRs interact with their respective HLA molecules (KIR-ligands; refer to Supplementary Tables S3 and S4) present on the tumor [13, 31]. In previous reports [12, 14, 32, 33], NK-based immunotherapies resulted in improved clinical outcome for patients with a KIR-ligand missing genotype as compared to those with a KIR-ligand present genotype. “KIR-ligand present” is defined as all KIR-ligands present for each inhibitory KIR gene present, whereas “KIR-ligand missing” is defined as having at least one KIR-ligand absent for the inhibitory KIR genes present (Supplementary Table S3). We have previously presented data that did not show improved outcome for patients with KIR-ligand-missing in specific studies for follicular lymphoma patients treated with maintenance rituximab, neuroblastoma patients treated with anti-GD2 dinutuximab, and metastatic renal cell carcinoma patients treated with HD-IL2 [9, 11, 34]. Here, we found no associations for KIR-ligand present/missing status among the IO-naïve cohort. For patients with a KIR-ligand missing genotype (*n* = 122 or 121), there was no significant improvement in PFS, OR, or tumor shrinkage as compared to patients with KIR-ligand present (*n* = 78 or 76) (Fig. 1A–C).

### Triple-negative breast cancer patients may have greater clinical benefit when all KIR-ligands are present

**Fig. 2:**
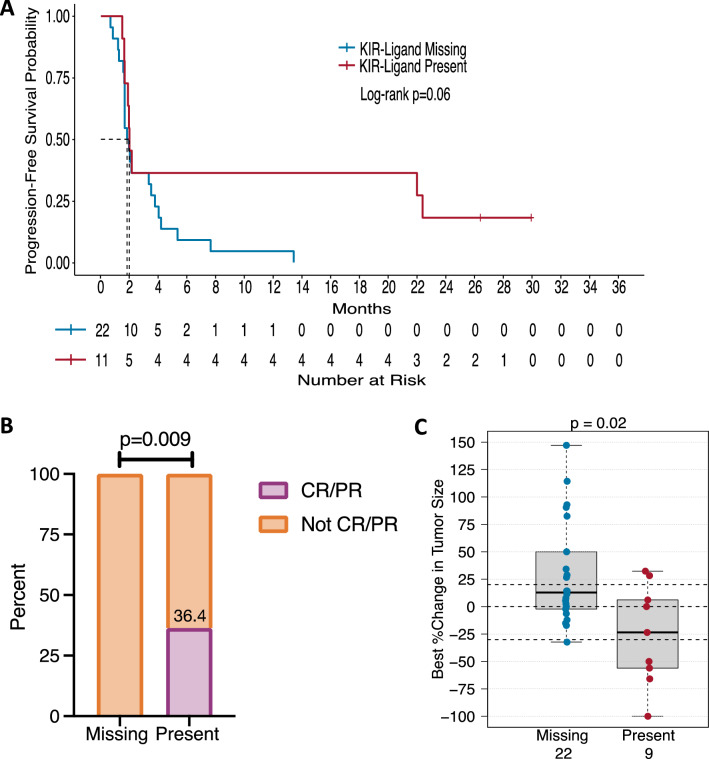
TNBC patients with a KIR/KIR-ligand present genotype have improved clinical response to BEMPEG plus nivolumab therapy. Kaplan–Meier curve for PFS (**a**) compares patients with a KIR-ligand missing genotype (blue line) to patients with a KIR-ligand present genotype (red line). OR is represented in (**b**) by the proportion of patients with a CR/PR (purple) compared to patients without a CR/PR (orange) based upon their KIR/KIR-ligand present/missing status. The box plots in (**c**) compare tumor shrinkage as indicated by the percent change in target lesion size from baseline for patients with a KIR-ligand missing genotype (blue) to patients with a KIR-ligand present genotype (red). Dotted horizontal lines indicate a +20% increase and −30% decrease in target lesion size from baseline

Although we observed no associations among the overall IO-naïve cohort for KIR-ligand present/missing status, we did observe that KIR-ligand present/missing status influenced clinical outcome in the triple-negative breast cancer (TNBC) patients (*n* = 33 or 31). Treatment with BEMPEG plus nivolumab showed a trend toward increased PFS for these KIR-ligand present TNBC patients (*p* = 0.06) (Fig. 2A) and significantly improved OR (*p* = 0.009; 36.4 vs. 0%) and tumor shrinkage (*p* = 0.02; median change −23.5 vs. +12.8%) as compared to their KIR-ligand missing counterparts (Fig. 2B, C).

### KIR2DL2 in the presence of its HLA-C1 ligand is associated with clinical benefit

The KIR-ligand present/missing analysis takes all three KIR-ligands (HLA-C1, HLA-C2, and HLA-Bw4) into consideration as contributing equally to the inhibition or education of NK cells. However, with no associations found for the overall group of 200 IO-naïve patients when assessing all inhibitory KIR/KIR-ligands simultaneously, we sought to investigate whether specific inhibitory KIR/KIR-ligand pairs may have an influence on clinical outcome with BEMPEG plus nivolumab combination therapy. Thus, we individually assessed the inhibitory KIRs in the presence or absence of their ligands.

KIR2DL2 is an inhibitory KIR that recognizes the HLA-C1 ligand. Compared to KIR2DL3, which shares the same ligand, KIR2DL2 has a stronger affinity for the HLA-C1 ligand [35]. Therefore, we assessed the patients who have KIR2DL2 in the presence of its HLA-C1 ligand (KIR2DL2+/HLA-C1+; *n* = 77) compared to the patients who did not have this specific receptor-ligand pair (***not*** KIR2DL2 + /HLA-C1+; *n* = 123 or 120) (Supplementary Table S4).

**Fig. 3:**
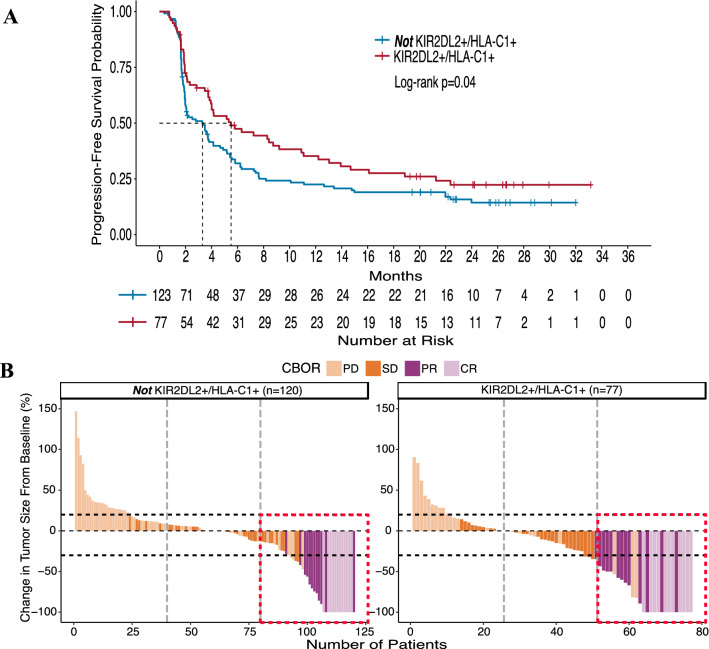
KIR2DL2+/HLA-C1+ patients have significantly greater clinical benefit compared to patients who are not KIR2DL2+/HLA-C1+. Kaplan–Meier curve for PFS **(a)** compares patients who are KIR2DL2+/HLA-C1+ (red line) to patients who are ***not*** KIR2DL2+/HLA-C1+ (blue line). Waterfall plots displaying OR and tumor shrinkage (**b)** compares patients who are KIR2DL2+/HLA-C1+ (right) with patients who are ***not*** KIR2DL2+/HLA-C1+ (left). CBOR is the confirmed best overall response by RECIST 1.1 criteria; PD, progressive disease (light orange); SD, stable disease (orange); PR, partial response (purple); CR, complete response (light purple). Vertical dotted lines divide the number of patients into thirds, and horizontal dotted lines indicate a +20% increase and −30% decrease in target lesion size from baseline. The dotted red box outlines the top third of patients, indicating a larger proportion of patients with a positive clinical response in the KIR2DL2+/HLA-C1+ group (right) than in the group who are ***not*** KIR2DL2+/HLA-C1+ (left)

Patients who were KIR2DL2+/HLA-C1+ showed significantly prolonged PFS (median 5.5 months vs. 3.3 months; *p* = 0.04) as compared to patients who were ***not*** KIR2DL2+/HLA-C1+ (Fig. 3A). To visually represent the clinical benefit seen from this genotype at an individual patient level, the waterfall plots (Fig. 3B) display the percent change in target lesion size from baseline for each patient along with their CBOR. Patients represented in the right waterfall plot (KIR2DL2+/HLA-C1+) had significantly greater tumor shrinkage (median change −13.0 vs. 0%; *p* = 0.01) as compared to the patients represented in the left waterfall plot (***not*** KIR2DL2+/HLA-C1+). Those same patients (KIR2DL2+/HLA-C1+) also showed a trend toward increased OR rate as indicated by the dotted red box representing a greater proportion of the top third percent of patients (31.2% with a CR/PR; right) having a CR/PR as compared to patients who were ***not*** KIR2DL2+/HLA-C1+ (19.5% with a CR/PR; left) (*p* = 0.07).

### KIR3DL1 and its HLA-Bw4 ligand do not influence clinical outcome

We also assessed the patients who have KIR3DL1 in the presence of its HLA-Bw4 ligand (KIR3DL1+/HLA-Bw4+; *n* = 59 or 58) compared to the patients who did not have this specific receptor-ligand pair (***not*** KIR3DL1+/HLA-Bw4+; *n* = 141 or 139) (Supplementary Table S4). In contrast with some previous reports where the inhibitory KIR3DL1 gene in the presence of its HLA-Bw4 ligand was associated with improved clinical outcome from immunotherapy [9, 11], here, we found no association of KIR3DL1/HLA-Bw4 with clinical response in patients receiving BEMPEG plus nivolumab combination therapy (Supplementary Figure 1A, B).

### Inhibitory KIR2DL2/HLA-C1+ interactions in combination with KIR3DL1/HLA-Bw4+ interactions improve outcome for patients receiving BEMPEG

Although we did not observe any influence of KIR3DL1 and its HLA-Bw4 ligand on clinical outcome (Supplementary Figure 1A, B), we investigated whether KIR3DL1 in combination with KIR2DL2 and their ligands could further influence patient outcomes. We have previously shown that this interaction of inhibitory KIR2DL2 and KIR3DL1 with their ligands was associated with improved outcome in follicular lymphoma and neuroblastoma patients receiving rituximab maintenance therapy and dinutuximab monoclonal antibody (mAb) immunotherapy, respectively [9, 11]. Thus, we compared the group of patients who had KIR2DL2 with its HLA-C1 ligand as well as KIR3DL1 with its HLA-Bw4 ligand (Group 2: KIR2DL2+/HLA-C1+ ***and*** KIR3DL1+/HLA-Bw4+; *n* = 52) to the remaining patients, i.e., those either lacking KIR2DL2, HLA-C1, KIR3DL1, and/or HLA-Bw4 (Group 1: ***not*** KIR2DL2+/HLA-C1+ ***and*** KIR3DL1+/HLA-Bw4+; *n* = 148 or 145). The distinct genotypes comprising these two groups are detailed in Supplementary Table S4.

**Fig. 4:**
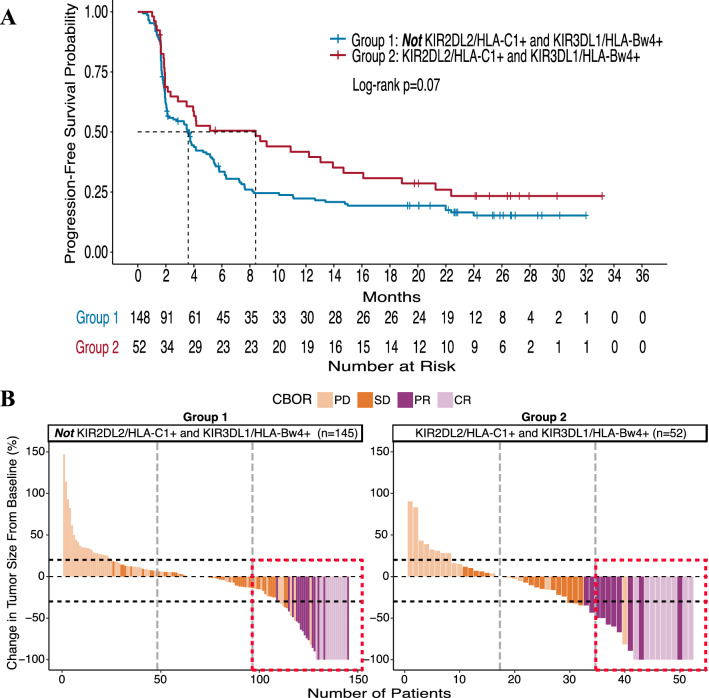
IO-naïve patients observe greater clinical benefit with a KIR2DL2+/HLA-C1+ and KIR3DL1+/HLA-Bw4+ genotype. Kaplan–Meier curve for PFS **(a)** compares patients who are KIR2DL2+/HLA-C1+ and KIR3DL1+/HLA-Bw4+ (Group 2, red line) to patients who are ***not*** KIR2DL2+/HLA-C1+ and KIR3DL1+/HLA-Bw4+ (Group 1, blue line). Waterfall plots displaying OR and tumor shrinkage (**b)** compares patients who are KIR2DL2+/HLA-C1+ and KIR3DL1+/HLA-Bw4+ (Group 2, right) with patients who are ***not*** KIR2DL2+/HLA-C1+ and KIR3DL1+/HLA-Bw4+ (Group 1, left). CBOR is the confirmed best overall response by RECIST 1.1 criteria; PD, progressive disease (light orange); SD, stable disease (orange); PR, partial response (purple); CR, complete response (light purple). Vertical dotted lines divide the number of patients into thirds, and horizontal dotted lines indicate a + 20% increase and −30% decrease in target lesion size from baseline. The dotted red box outlines the top third of patients, indicating a larger proportion of patients with a positive clinical response in Group 2 (right) than in Group 1 (left)

We found that patients who were in Group 2 had significantly improved OR and tumor shrinkage, *p* = 0.02 and *p* = 0.04, respectively. Although not significant, Group 2 patients also showed a trend toward prolonged PFS, *p* = 0.07 (Fig. 4A). Visualized at the individual patient level, the waterfall plots of the percent change in target lesion size from baseline (Fig. 4B) demonstrate that within the Group 2 genotype (right), a greater proportion of the top third percent of patients, outlined in the dotted red box, had a CR or PR (19/52 patients with CR/PR vs. 29/148) along with greater tumor shrinkage (median change −16.1% vs. 0%) compared to Group 1 (left).

### Overall associations of KIR/KIR-ligand genotypes with outcome for the specific groups of cancer patients tested

The specific genotype groupings shown for the overall 200 IO-naïve patients shown in Figs. 1, 3, 4 and S1 are shown for each of the separate disease groups tested, in Supplementary Table S5A-D. In addition to the association of KIR-ligands present with improved outcome for TNBC patients, shown above in Fig. 2, we note that the KIR2DL2/HLA-C1 associations (Supplementary Table S5B) are conserved in some of the clinical parameters for metastatic urothelial cancer (mUC; OR *p* = 0.05, tumor shrinkage *p* = 0.003) and non-small cell lung cancer (NSCLC; tumor shrinkage *p* = 0.05). Similar significance is observed in the KIR2DL2/HLA-C1 ***and*** KIR3DL1/HLA-Bw4 associations (Supplementary Table S5D) for mUC (OR *p* = 0.02, tumor shrinkage *p* = 0.005) as well. Although the primary results for all cohorts have been presented in various forms, not all cohorts have had the primary results published. Additionally, it is worth noting the small patient populations that these results are comprised of when analyzed as distinct cohorts. Thus, these results for the separate disease groups should be interpreted with caution due to multiplicity of testing without adjustment until primary results have been published for all cohorts.

### SNPs among FCγR genes (2A, 2C, 3A) were not associated with clinical outcome

In our prior report for metastatic renal cell carcinoma patients treated with HD-IL2, we observed that improved clinical response may be correlated with specific “high-affinity” SNPs within the three FCγR genes assessed here, including FCγR2A, 2C, and 3A [10]. However, in this study, we did not find any significant associations between FCγR and clinical outcome (Supplementary Tables S6 and S7).

## Discussion

In this retrospective analysis of patients with advanced solid tumors who received BEMPEG plus nivolumab combination therapy as part of the PIVOT-02 trial, we assessed the potential associations of KIR/KIR-ligand genotypes with clinical outcome. Here, we show that the repertoire of KIR/KIR-ligands that an individual inherits is associated with their clinical response to BEMPEG plus nivolumab treatment; consistent with a role for NK cells in the anti-tumor efficacy of this combination therapy.

Unlike some prior reports [12, 14, 32, 33], we found no evidence of improved clinical response for IO-naïve patients with the KIR-ligands missing genotype compared to the KIR-ligands present genotype (Fig. 1). Interestingly, however, we did find that for TNBC patients, response to BEMPEG plus nivolumab combination therapy was influenced by their KIR-ligand present/missing status. Namely, those with KIR-ligands present had greater clinical benefit in both OR and tumor shrinkage and a trend toward prolonged PFS as compared to those with KIR-ligands missing (Fig. 2). TNBC has been shown to be susceptible to NK cell-induced lysis supporting a role for NK cells in the anti-tumor response toward TNBC [36]. Yet, these prior reports [12, 14, 32, 33] mentioned above have shown the KIR-ligand missing genotype as beneficial for patients’ responses, in part due to NK-mediated killing of tumor cells under conditions in which the inhibitory KIRs on the NK cells are not interacting with their corresponding inhibitory ligands on the tumor cells, thus being less inhibited [13]. In the case of tumors that have no or low expression of KIR-ligands (namely HLA Class I), KIR-mediated inhibition would not be relevant, as the KIRs would not be seeing their corresponding ligand. This may be the case for TNBC, which has recently been shown to have prominent HLA-I loss, with more than half of patients having subclonal or diffuse HLA-I loss [26]. In this setting, the impact of NK cell licensing should also be taken into consideration.

The interactions of the KIRs on the NK cells with their corresponding HLA ligands on healthy cells during NK development have been shown to be associated with greater downstream NK cell function as a result of licensing [37–40]. If these properly licensed NK cells become activated through immune stimulatory signaling (i.e., through BEMPEG plus nivolumab), they can readily kill non-HLA-expressing tumor cells. In Wang et al., we previously reported that KIR/KIR-ligand genotypes known to directly drive licensing of NK cells can influence the functionality of NK cells following ex vivo immune stimulatory signaling when expanding NK cells [41]. Here, the combination treatment of BEMPEG and nivolumab might provide similar immune stimulatory signaling on endogenously licensed NK cells in the TME. Therefore, we hypothesize that patients with the KIR-ligands present genotype may have more ‘licensed’ (namely more potent) NK cells that, in this setting, would not be inhibited due to the low level of HLA on their tumors and thus be associated with a greater anti-tumor effect. In addition to TNBC, HLA-I downregulation or loss has been observed in 40–90% of human tumors, including those tumor types enrolled in this trial (melanoma, urothelial, NSCLC, and RCC) [42–48]. Thus, when combined with an immunotherapy regimen that activates NK cell activity and function (BEMPEG) together with an agent that represses checkpoint inhibition (nivolumab), in patients with cancers that have low or no HLA-I expression, these licensed NK cells may elicit enhanced tumor killing. In this scenario for patients with KIR-ligands present, this effective immunotherapy combination of BEMPEG plus nivolumab may take advantage of these licensed NK cells to overcome the inhibitory signaling from KIR/KIR-ligand interactions that may be present within the TME, such that NK cells can have persistent responses to reduce the tumor burden. Thus, our findings in the TNBC cohort may indicate a role for licensed NK cells in the anti-tumor response against TNBC following BEMPEG plus nivolumab therapy.

The impact of NK cell licensing may further explain our findings when we individually assessed the inhibitory KIRs in the presence of their ligands. For the inhibitory KIR2DL2 and its HLA-C1 ligand, patients who inherited both (KIR2DL2+/HLA-C1+) observed greater clinical responses (significant for PFS and tumor shrinkage and a trend for OR) as compared to the responses seen in the complementary group of patients (***not*** KIR2DL2+/HLA-C1+) (Fig. 3). Conversely, we did not see similar associations with the inhibitory KIR3DL1 and its HLA-Bw4 ligand when assessed alone (Supplementary Figure S1). When we further assessed the potential interactions between these two inhibitory KIRs simultaneously, we found that patients who inherited both KIRs in the presence of their ligands (Group 2: KIR2DL2+/HLA-C1+ ***and*** KIR3DL1+/HLA-Bw4+) had greater clinical benefit (significant for OR and tumor shrinkage and a trend for PFS) as compared to the complementary group of patients (Group 1: ***not*** KIR2DL2+/HLA-C1+ ***and*** KIR3DL1+/HLA-Bw4+) (Fig. 4). In separate randomized studies of anti-GD2 mAb and rituximab maintenance immunotherapy for neuroblastoma and follicular lymphoma patients, respectively, we found similar results. Namely, those patients with a Group 2 genotype had improved outcome from the anti-GD2 or rituximab maintenance immunotherapy (vs. no immunotherapy for neuroblastoma or non-maintenance for follicular lymphoma) [9, 11]. Altogether, these similar findings, which include different patient populations and disease types, provide some degree of validation for the influence of KIR/KIR-ligand genotypes on clinical outcome with immunotherapy regimens. Yet, the associations found in the IO-naïve patients in the PIVOT-02 trial need further evaluation prior to any clinical application, especially considering the limitation of the single-arm nature of this trial.

The associations of KIR/KIR-ligand genotypes with clinical outcome in the subset of IO-naïve patients in this trial suggest that either the immunologic effect of BEMPEG, or of nivolumab, or of their combined effect, may involve NK function in a way that influences outcome in this group of patients with solid tumors receiving this combination regimen. A prior study has reported that there was no association of KIR genotype, nor of KIR/KIR-ligand genotype with outcome in a group of 112 patients with advanced melanoma treated with single agent nivolumab [49]. This suggests, but does not prove, that the associations of KIR/KIR-ligand genotype with outcome observed here may be due to the effect of BEMPEG or due to an interaction of BEMPEG and nivolumab, rather than due to the action of nivolumab alone. Nivolumab and BEMPEG should synergize to drive a persistent anti-tumor response, via activation of immune cells (BEMPEG) while overcoming inhibitory signaling (nivolumab). NK cells primarily express CD122/CD132 (IL2βγ receptor). Since BEMPEG was designed to preferentially bind to this IL2βγ receptor (as compared to the IL2αβγ receptor found on Tregs), it should lead to NK cell activation and enhanced expansion of NK cells and CD8 T cells compared to Tregs. Yet, recent findings by Hashimoto and colleagues suggest that the ability of IL-2 to interact with CD25 (IL2α receptor) may influence the efficacy of IL-2 therapies given in combination with anti-PD-1. Data showed that with a mutated version of IL-2 that does not bind to CD25 (like BEMPEG), the synergy between IL-2 cytokine and anti-PD-1 (like nivolumab) treatment was abrogated [50]. Furthermore, upon activation, NK cells can also express CD25 [51]. This can increase NK cells’ affinity for IL-2, thereby enhancing their cytotoxic capabilities but also allowing NK cells to compete with Tregs for available IL-2 [52, 53]. Thus, the potential role that NK cells and KIR/KIR-ligand genotypes have in influencing response to IL-2 therapeutic combinations, including anti-PD-1 nivolumab, may be dependent on how that IL-2 therapy can bind to the IL2αβγ versus the IL2βγ receptors.

In the aforementioned studies for neuroblastoma and follicular lymphoma patients, both were two-arm, randomized clinical trials which allowed us to further suggest, based upon the results, a potential to discriminate between patients who are likely to benefit from anti-GD2 mAb or rituximab maintenance immunotherapy and those who are not. Such findings, once validated in the respective disease/treatment settings, may enable these genotypes to be used as predictive biomarkers to select which patients are most likely to benefit from immunotherapy [54]. This type of genotype-based patient selection for therapy may have greater clinical application, as many of the standard immunotherapy regimens are accompanied by toxic side effects. Thus, the ability to discern which patients may benefit from immunotherapy will in turn limit the number of patients we subject to these toxic regimens. In contrast, all patients evaluated in this current non-randomized PIVOT-02 trial received the combination therapy of BEMPEG and nivolumab. Therefore, the genotypes we identified as associated with improved outcome, if validated, would potentially be prognostic of improved outcome among patients receiving this combination therapy.

In summary, our evaluation of these immunogenotypes suggests that the repertoire of KIR/KIR-ligands that an individual inherits is associated with their clinical response to BEMPEG plus nivolumab therapy among those who are IO-naïve in the PIVOT-02 trial, which further supports a role for NK cells in the anti-tumor efficacy of this combination therapy. Further clinical validation in other studies is needed to determine whether these immunogenotypes may provide prospective data that could be clinically actionable.

## Supplementary Information

Below is the link to the electronic supplementary material.

Supplementary file1 (PDF 988 kb)

## Abbreviations

ADCC: Antibody-dependent cellular cytotoxicity
BEMPEG: Bempegaldesleukin
CBOR: Confirmed best overall response
CR: Complete response
FCγRs: Fc-gamma receptors
Fc: Fragment crystallizable
HD-IL2: High dose IL2
ICIs: Immune checkpoint inhibitors
IO-naïve: Immunotherapy naïve
KIRs: Killer immunoglobulin-like receptors
mAb: Monoclonal antibody
mUC: Metastatic urothelial cancer
NSCLC: Non-small cell lung cancer
OR: Objective response
ORR: Objective response rate
PD: Progressive disease
PD-1: Anti-programmed death-1
PFS: Progression-free survival
PR: Partial response
RP2D: Recommended phase 2 dose
SD: Stable disease
SNPs: Single-nucleotide polymorphisms
SSP-PCR: Sequence specific primers-polymerase chain reaction
TME: Tumor microenvironment
TNBC: Triple-negative breast cancer
Tregs: T regulatory cells
TS: Tumor shrinkage
