# Supplementary material for: Associations between KIR/KIR-ligand genotypes and clinical outcome for patients with advanced solid tumors receiving BEMPEG plus nivolumab combination therapy in the PIVOT-02 trial
Source: Cancer Immunol Immunother. 2023 Feb 23;72(7):2099–111. doi: 10.1007/s00262-023-03383-w (PMC10264535; doi:10.1007/s00262-023-03383-w)
Supplement: Supplementary file 1 — Supplementary file1 (PDF 988 kb) [file 262_2023_3383_MOESM1_ESM.pdf]

# Supplementary Table S1

| RP2D (n=213) | Genotype | ORR | | | PFS | | Tumor Shrinkage | |
|---|---|---|---|---|---|---|---|---|
| | | n | CR/PR | P | Median (months, 95% CI) | P | Median (IQR, n) | P |
| KIR/KIR-ligand | Present | 19/83 | 22.9% | 0.97 | 3.9 (2.1-5.6; n=83; e=65) | 0.54 | -1.4 (-34.8 to 13.0, n = 81) | 0.7 |
| | Missing | 30/130 | 23.1% | | 3.7 (2.3-4.9; n=130; e=103) | | -2.1 (-37.9 to 11.8, n = 129) | |
| | +/+ | 25/85 | 29.4% | 0.08 | 4.2 (3.7-8.3; n=85; e=62) | 0.06 | -10.5 (-57.7 to 6.5, n = 85) | 0.04 |
| KIR2DL2/C1 | not +/+ | 24/128 | 18.8% | | 3.3 (2-3.8; n=128; e=106) | | 0 (-24.2 to 12.8, n = 125) | |
| | +/+ | 35/151 | 23.2% | 0.92 | 3.5 (2.1-4.7; n=151; e=121) | 0.41 | -1 (-35.3 to 12.2, n = 149) | 0.44 |
| KIR3DL1/Bw4 | not +/+ | 14/62 | 22.6% | | 4.0 (2.1-6.3; n=62; e=47) | | -3.8 (-47.2 to 11.3, n = 61) | |
| KIR2DL2/C1 and | +/+ | 20/59 | 33.9% | 0.03 | 4.2 (2.3-10.9; n=59; e=43) | 0.11 | -13.0 (-63.5 to 8.6, n = 59) | 0.11 |
| KIR3DL1/Bw4 | not +/+ | 29/154 | 18.8% | | 3.6 (2.1-4.1; n=154; e=125) | | 0 (-25.0 to 12.2, n = 151) | |

**Table S1: Four associations of KIR/KIR-ligand genotypes with outcome for the RP2D cohort of patients.** For the RP2D cohort (n=213), including the 13 patients who had prior IO treatment, the associations of KIR-ligand present vs missing status (first row), KIR2DL2/HLA-C1 genotype (second row), KIR3DL1/HLA-Bw4 genotype (third row), and KIR2DL1/HLA-C1 *and* KIR3DL1/HLA-Bw4 genotype (fourth row) with the three clinical outcome parameters (OR, PFS, and tumor shrinkage) are shown. The definitions for the genotype categories are detailed in Supplemental Table S3 and S4. Abbreviations: RP2D, recommended phase II dose; CI, confidence interval; IQR, interquartile range.

## Supplementary Table S2

**A**

| KIR and KIR-Ligand Gene Status | % Genotype Presence |
|---|---|
| KIR2DL1 | 97 |
| KIR2DL2 | 44 |
| KIR2DL3 | 93 |
| KIR3DL1 | 97 |
| HLA-C1/C1 | 38 |
| HLA-C1/C2 | 51 |
| HLA-C2/C2 | 11 |
| HLA-Bw4 | 73 |
| KIR-ligands Present | 39 |
| KIR-ligands Missing | 61 |

**B**

| | FCGR SNP Gene Status | % Genotype Presence |
|---|---|---|
| **FCGR2A** | H/H | 26 |
| | H/R | 49 |
| | R/R | 25 |
| **FCGR3A** | V/V | 7 |
| | V/F | 44 |
| | F/F | 49 |
| **FCGR2C** | C/C | 3 |
| | C/T | 27 |
| | T/T | 71 |

**Table S2: KIR/KIR-ligand and FCGR SNP genotype frequencies for patients analyzed within the IO-naïve cohort.** For each indicated KIR and KIR-ligand designation (A) and FCGR SNP status (B) shown, the frequency of patients in this genotyped population of 200 IO-naïve patients is shown. The definition of KIR-ligands present and KIR-ligand missing is detailed in Supplemental Table S3.

## Supplementary Table S3

| KIR-ligands present genotypes n=78 | KIR-ligand missing genotypes n=122 |
|---|---|
| KIR2DL1+ with KIR2DL2 and/or KIR2DL3+, C1/C2, with KIR3DL1+, Bw4+ | KIR2DL1+ with KIR2DL2 and/or KIR2DL3+, C1/C1, with KIR3DL1+, Bw4+ or Bw4- |
| KIR2DL1- with KIR2DL2 and/or KIR2DL3+, C1/C1 or C1/C2, with KIR3DL1+, Bw4+ | KIR2DL1+ with KIR2DL2 and/or KIR2DL3+, C1/C2, with KIR3DL1+, Bw4- |
| KIR2DL1+ with KIR2DL2 and/or KIR2DL3+, C1/C2, with KIR3DL1-, Bw4+ or Bw4- | KIR2DL1+ with KIR2DL2 and/or KIR2DL3+, C2/C2, with KIR3DL1+, Bw4+ or Bw4- |
| KIR2DL1- with KIR2DL2 and/or KIR2DL3+, C1/C1 or C1/C2, with KIR3DL1-, Bw4+ or Bw4- | |

**Table S3: KIR/KIR-ligand genotypes that determine KIR-ligand present/missing status.** For this study, we defined individuals to be all KIR-ligands present if they fulfill all of the following criteria: (1) if KIR2DL1+, must have HLA-C2; (2) must have HLA-C1 (as all individuals will be positive for either, or both, KIR2DL2 and KIR2DL3); and (3) if KIR3DL1+, must have HLA-Bw4. An individual is missing at least one KIR-ligand if they fulfill any of the following criteria: (1) if KIR2DL1+ and missing the HLA-C2; and/or (2) if missing the HLA-C1 motif; and/or (3) if KIR3DL1+, and they are negative for HLA-Bw4.

## Supplementary Table S4

| KIR2DL2/KIR-ligand genotypes | | KIR3DL1/KIR-ligand genotypes | |
|---|---|---|---|
| **KIR2DL2+/C1+**<br>n=77 | ***Not* KIR2DL2+/C1+**<br>n=123 | **KIR3DL1+/Bw4+**<br>n=59 | ***Not* KIR3DL1+/Bw4+**<br>n=141 |
| KIR2DL2+, HLA-C1+ | KIR2DL2+, HLA-C1- | KIR3DL1+, HLA-Bw4+ | KIR3DL1+, HLA-Bw4- |
| | KIR2DL2-, HLA-C1+ | | KIR3DL1-, HLA-Bw4+ |
| | KIR2DL2-, HLA-C1- | | KIR3DL1-, HLA-Bw4- |

| KIR2DL2/KIR-ligand WITH KIR3DL1/KIR-ligand genotypes | | |
|---|---|---|
| **Group 1:**<br>**KIR2DL2+/C1+ AND KIR3DL1+/Bw4+**<br>n=52 | **Group 2:**<br>***Not* KIR2DL2+/C1+ AND KIR3DL1+/Bw4+**<br>n=148 | |
| KIR2DL2+, HLA-C1+, KIR3DL1+, HLA-Bw4+ | KIR2DL2+, HLA-C1+, KIR3DL1-, HLA-Bw4+ or - | KIR2DL2-, HLA-C1+, KIR3DL1+ or -, HLA-Bw4+ or - |
| | KIR2DL2+, HLA-C1+, KIR3DL1+, HLA-Bw4- | KIR2DL2+ or -, HLA-C1-, KIR3DL1+ or -, HLA-Bw4+ or - |

**Table S4: KIR/ligand pair genotype categories discussed in manuscript.** KIR2DL2/ligand and KIR3DL1/ligand genotypes that compose the KIR2DL2+/HLA-C1+ vs. *not* KIR2DL2+/HLA-C1+ (Figure 3); KIR3DL1+/HLA-Bw4+ vs. *not* KIR3DL1+/HLA-Bw4+ (Supplemental Figure 1); and KIR2DL2+/HLA-C1+ *and* KIR3DL1+/HLA-Bw4+ (Group 2) vs. *not* KIR2DL2+/HLA-C1+ *and* KIR3DL1+/HLA-Bw4+ (Group 1) (Figure 4) genotype categories. For each genotype category mentioned, the columns show the relevant KIR and KIR-ligand genotype possibilities that comprise the category. At the top of each column, the number of patients in this study with that genotype are also shown. For each of the 3 main columns, those with and those *not* with the indicated genotype add up to the total of 200 patients in this study.

# Supplementary Table S5

**A**

| | | Genotype | ORR | | | PFS | | Tumor Shrinkage | |
|---|---|---|---|---|---|---|---|---|---|
| **Cohort** | **N** | **KIR/KIR-ligand** | **n** | **CR/PR** | **P** | **Median (months, 95% CI)** | **P** | **Median (IQR, n)** | **P** |
| **Melanoma** | 31 | Present | 3/10 | 30.0% | 0.17 | NR (1.1-NR; n=10; e=4) | 0.9 | -13 (-100 to 13, n = 10) | 0.51 |
| | | Missing | 11/21 | 52.4% | | 8.2 (2-NR; n=21; e=10) | | -67 (-100 to 0, n = 21) | |
| **Renal Cell Carcinoma** | 37 | Present | 4/15 | 26.7% | 0.97 | 10.1 (1.6-22.4; n=15; e=11) | 0.46 | 3.6 (-22.8 to 8.6, n = 15) | 0.5 |
| | | Missing | 6/22 | 27.3% | | 5.3 (3.5-12.2; n=22; e=18) | | 0 (-54.1 to 6.4, n = 22) | |
| **Metastatic Urothelial Cancer** | 37 | Present | 6/15 | 40.0% | 0.58 | 4.1 (1.6-13; n=15; e=12) | 0.91 | -35.3 (-100 to -4.8, n = 15) | 0.47 |
| | | Missing | 7/22 | 31.8% | | 4.1 (2.1-12.6; n=22; e=17) | | -16.1 (-53.4 to -2.1, n = 22) | |
| **Triple Negative Breast Cancer** | 33 | Present | 4/11 | 36.4% | 0.009 | 2.0 (1.6-22.4; n=11; e=9) | 0.06 | -23.5 (-56.1 to 6.1, n = 9) | 0.02 |
| | | Missing | 0/22 | 0.0% | | 1.9 (1.6-3.5; n=22; e=22) | | 12.8 (-2.2 to 50, n = 22) | |
| **Non-Small Cell Lung Cancer** | 21 | Present | 1/8 | 12.5% | 0.53 | 4.4 (1.3-8.4; n=8; e=7) | 0.73 | -1.3 (-11.7 to 3.4, n = 8) | 0.23 |
| | | Missing | 3/13 | 23.1% | | 3.7 (1.7-11.1; n=13; e=10) | | -21.1 (-33.1 to 0, n = 13) | |
| **IO-naïve** | | **Present** | **19/78** | **24.4%** | **0.92** | **3.6 (2-5.8; n=78; e=60)** | **0.63** | **-4.8 (-39.4 to 12.4, n = 76)** | **0.66** |
| | | **Missing** | **29/122** | **23.8%** | | **3.7 (2.8-5.3; n=122; e=96)** | | **-3.1 (-42.0 to 9.1, n = 121)** | |

**B**

| | | Genotype | ORR | | | PFS | | Tumor Shrinkage | |
|---|---|---|---|---|---|---|---|---|---|
| **Cohort** | **N** | **KIR2DL2/C1** | **n** | **CR/PR** | **P** | **Median (months, 95% CI)** | **P** | **Median (IQR, n)** | **P** |
| **Melanoma** | 31 | +/+ | 4/9 | 44.4% | 0.95 | NR (1.1-NR; n=9; e=3) | 0.58 | -22 (-100 to 15, n = 9) | 0.62 |
| | | not +/+ | 10/22 | 45.5% | | 8.2 (2-NR; n=22; e=11) | | -25.5 (-100 to 0, n = 22) | |
| **Renal Cell Carcinoma** | 37 | +/+ | 6/19 | 31.6% | 0.5 | 9.2 (3.5-16.1; n=19; e=15) | 0.91 | -10.5 (-56.3 to 4.1, n = 19) | 0.12 |
| | | not +/+ | 4/18 | 22.2% | | 5.8 (2.1-10.1; n=18; e=14) | | 5.5 (-37.9 to 11.8, n = 18) | |
| **Metastatic Urothelial Cancer** | 37 | +/+ | 7/13 | 53.8% | 0.05 | 10.9 (1.6-18.9; n=13; e=10) | 0.66 | -81.6 (-100 to -31.6, n = 13) | 0.003 |
| | | not +/+ | 6/24 | 25.0% | | 3.5 (1.9-6.2; n=24; e=19) | | -13.3 (-38.7 to -0.9, n = 24) | |
| **Triple Negative Breast Cancer** | 33 | +/+ | 2/10 | 20.0% | 0.42 | 3.9 (1.6-5.3; n=10; e=8) | 0.07 | 0.2 (-32.4 to 28.2, n = 10) | 0.35 |
| | | not +/+ | 2/23 | 8.7% | | 1.8 (1.6-2.0; n=23; e=23) | | 7.7 (-6.4 to 34.2, n = 21) | |
| **Non-Small Cell Lung Cancer** | 21 | +/+ | 3/12 | 25.0% | 0.41 | 5.8 (1.9-NR; n=12; e=8) | 0.31 | -18.7 (-34.2 to -6.2, n = 12) | 0.05 |
| | | not +/+ | 1/9 | 11.1% | | 3.6 (1.7-7.4; n=9; e=9) | | 1.1 (-1.2 to 5.7, n = 9) | |
| **IO-naïve** | | **+/+** | **24/77** | **31.2%** | **0.06** | **5.5 (3.7-9.2; n=77; e=55)** | **0.04** | **-13.0 (-60 to 4.5, n = 77)** | **0.01** |
| | | **not +/+** | **24/123** | **19.5%** | | **3.3 (2-4.1; n=123; e=101)** | | **0 (-26.8 to 12.2, n = 120)** | |

**C**

| | | Genotype | ORR | | | PFS | | Tumor Shrinkage | |
|---|---|---|---|---|---|---|---|---|---|
| **Cohort** | **N** | **KIR3DL1/Bw4** | **n** | **CR/PR** | **P** | **Median (months, 95% CI)** | **P** | **Median (IQR, n)** | **P** |
| **Melanoma** | 31 | +/+ | 9/22 | 40.9% | 0.39 | 8.2 (2-NR; n=22; e=11) | 0.46 | -18 (-100 to 13, n = 22) | 0.42 |
| | | not +/+ | 5/9 | 55.6% | | NR (1.6-NR; n=9; e=3) | | -100 (-100 to -4, n = 9) | |
| **Renal Cell Carcinoma** | 37 | +/+ | 5/23 | 21.7% | 0.33 | 7.3 (3.7-21.3; n=30; e=22) | 0.18 | 3.6 (-34.7 to 8.0, n = 23) | 0.3 |
| | | not +/+ | 5/14 | 35.7% | | 6.9 (2.1-NR; n=14; e=9) | | -5.5 (-63.8 to 5.4, n = 14) | |
| **Metastatic Urothelial Cancer** | 37 | +/+ | 11/29 | 37.9% | 0.42 | 4.1 (2.1-10.9; n=29; e=22) | 0.93 | -30 (-100 to -2.8, n = 29) | 0.47 |
| | | not +/+ | 2/8 | 25.0% | | 5.2 (1.9-14.8; n=8; e=7) | | -20.8 (-50.3 to -7.4, n = 8) | |
| **Triple Negative Breast Cancer** | 33 | +/+ | 4/24 | 16.7% | 0.2 | 1.7 (1.6-2.1; n=24; e=22) | 0.77 | 6.9 (-23.5 to 34.2, n = 22) | 1 |
| | | not +/+ | 0/9 | 0.0% | | 3.3 (1.2-5.3; n=9; e=9) | | 2.6 (-2.2 to 14.3, n = 9) | |
| **Non-Small Cell Lung Cancer** | 21 | +/+ | 3/14 | 21.4% | 0.68 | 3.7 (1.9-8.4; n=14; e=12) | 0.79 | -1.3 (-16.3 to 5.1, n = 14) | 0.16 |
| | | not +/+ | 1/7 | 14.3% | | 5.8 (1.7-NR; n=7; e=5) | | -23.7 (-29.0 to -14.0, n = 7) | |
| **IO-naïve** | | **+/+** | **34/141** | **24.1%** | **0.95** | **3.6 (2.1-4.9; n=141; e=112)** | **0.35** | **-1.4 (-37.5 to 11.9, n = 139)** | **0.35** |
| | | **not +/+** | **14/59** | **23.7%** | | **4.1 (2.1-7.2; n=59; e=44)** | | **-5 (-48.8 to 7.3, n = 58)** | |

**D**

| | | Genotype | ORR | | | PFS | | Tumor Shrinkage | |
|---|---|---|---|---|---|---|---|---|---|
| **Cohort** | **N** | **KIR2DL2/C1 and KIR3DL1/Bw4** | **n** | **CR/PR** | **P** | **Median (months, 95% CI)** | **P** | **Median (IQR, n)** | **P** |
| **Melanoma** | 31 | +/+ and +/+ | 2/6 | 33.3% | 0.42 | 1.9 (1.1=NR; n=6; e=3) | 0.56 | -3.5 (-67 to 16, n = 6) | 0.24 |
| | | not +/+ and +/+ | 12/25 | 48.0% | | NR (3.7-NR; n=25; e=11) | | -37 (-100 to 0, n = 25) | |
| **Renal Cell Carcinoma** | 37 | +/+ and +/+ | 4/12 | 33.3% | 0.53 | 12.2 (1.6-21.3; n=12; e=10) | 0.91 | -6.8 (-62.0 to 5.7, n = 12) | 0.53 |
| | | not +/+ and +/+ | 6/25 | 24.0% | | 5.5 (3.8-10.1; n=25; e=19) | | 0 (-48.8 to 7.3, n = 25) | |
| **Metastatic Urothelial Cancer** | 37 | +/+ and +/+ | 7/12 | 58.3% | 0.02 | 10.9 (1.6-18.9; n=12; e=9) | 0.48 | -90.8 (-100 to -23.7, n = 12) | 0.005 |
| | | not +/+ and +/+ | 6/25 | 24.0% | | 3.5 (2-6.2; n=25; e=20) | | -13.5 (-42.0 to -1.8, n = 25) | |
| **Triple Negative Breast Cancer** | 33 | +/+ and +/+ | 2/6 | 33.3% | 0.12 | 3.0 (1.6-NR; n=6; e=4) | 0.11 | -2.5 (-50 to 32.3, n = 6) | 0.57 |
| | | not +/+ and +/+ | 2/27 | 7.4% | | 1.9 (1.6-3.3; n=27; e=27) | | 6.1 (-6.4 to 28.8, n = 25) | |
| **Non-Small Cell Lung Cancer** | 21 | +/+ and +/+ | 2/7 | 28.6% | 0.42 | 2.9 (1.3-NR; n=7; e=5) | 0.66 | -7.1 (-29.9 to -3.5, n = 7) | 0.65 |
| | | not +/+ and +/+ | 2/14 | 14.3% | | 3.7 (1.7-7.4; n=14; e=12) | | -4.0 (-25 to 5.1, n = 14) | |
| **IO-naïve** | | **+/+** | **19/52** | **36.5%** | **0.02** | **8.4 (2.3-13.9; n=52; e=37)** | **0.07** | **-16.1 (-74.3 to 6.5, n = 52)** | **0.04** |
| | | **not +/+** | **29/148** | **19.6%** | | **3.6 (2.1-4.7; n=148; e=119)** | | **0 (-28.6 to 11.8, n = 145)** | |

**Table S5: Overall associations of KIR/KIR-ligand genotypes with outcome for the specific groups of cancer patients analyzed.** Associations of KIR-ligand present vs missing status (a), KIR2DL2/HLA-C1 genotype (b), KIR3DL1/HLA-Bw4 genotype (c), and KIR2DL1/HLA-C1 *and* KIR3DL1/HLA-Bw4 genotype (d) with the three clinical outcome parameters (OR, PFS, and tumor shrinkage) across the five cancer types analyzed. The definitions for the genotype categories are detailed in Supplemental Table S3 and S4. Abbreviations: CI, confidence interval; IQR, interquartile range; NR, reported where the median PFS is "not reached."

# Supplementary Table S6

| | | FCGR3A | | |
|---|---|---|---|---|
| | | V/V | V/F | F/F |
| FCGR2A | H/H | | | |
| | H/R | | | |
| | R/R | | | |

| | | FCGR3A | | |
|---|---|---|---|---|
| | | V/V | V/F | F/F |
| FCGR2C | C/C | | | |
| | C/T | | | |
| | T/T | | | |

| | | | | FCGR3A | | |
|---|---|---|---|---|---|---|
| | | | | V/V | V/F | F/F |
| FCGR2A | H/H | FCGR2C | C/C | | | |
| | | | C/T | | | |
| | | | T/T | | | |
| | | | | FCGR3A | | |
| | | | | V/V | V/F | F/F |
| | H/R | FCGR2C | C/C | | | |
| | | | C/T | | | |
| | | | T/T | | | |
| | | | | FCGR3A | | |
| | | | | V/V | V/F | F/F |
| | R/R | FCGR2C | C/C | | | |
| | | | C/T | | | |
| | | | T/T | | | |

**Table S6: FCγR genotype categories.** The three separate genotypes for FCγR3A (V/V, V/F, and F/F), when combined with the three separate genotypes for FCγR2A: H/H (top); H/R (middle); and R/R (bottom) yield nine separate genotypes. Here, these are combined with the three separate genotypes for FCγR2C (C/C, C/T, and T/T). When genotypes for all three of these loci are combined (27 separate boxes), we divided them into favorable (shaded) vs unfavorable (unshaded) genotypes. The favorable group includes all patients homozygous for H/H or V/V as well as patients expressing one copy of V and one copy of H and at least one copy of FCγR2C-‘C’. All others, namely those patients that do not have at least two copies of either high-affinity allele (F/F-R/R, V/F-R/R, or F/F-H/R), and those patients heterozygous for V/F and H/R but lacking any expression of FCγR2C, are unshaded and labeled as unfavorable.

# Supplementary Table S7

| | Genotype Group | ORR | | | PFS | | Tumor Shrinkage | |
|---|---|---|---|---|---|---|---|---|
| | | n | CR/PR | *P* | Median (months; 95% CI) | *P* | Median (IQR, n) | *P* |
| **FCGR2A SNP** | HH | 14/52 | 26.9% | 0.57 | 3.8 (2-7.7; n=52; e=41) | 0.99 | -5.6 (-58.9 to 8.3, n = 51) | 0.84 |
| | HR or RR | 34/148 | 23.0% | | 3.7 (2.2-5.2; n=148; e=115) | | -2.5 (-37.9 to 12.1, n = 146) | |
| **FCGR2C SNP** | CC or CT | 18/58 | 31.0% | 0.15 | 3.8 (1.9-7.3; n=58; e=45) | 0.82 | -6 (-70.6 to 7.7, n = 57) | 0.34 |
| | TT | 30/142 | 21.1% | | 3.7 (2.3-5.2; n=142; e=111) | | -2.6 (-35.0 to 11.6, n = 140) | |
| **FCGR3A SNP** | VV | 4/14 | 28.6% | 0.67 | 3.9 (1.7-14.7; n=14; e=11) | 0.96 | -4.4 (-60 to 5.4, n = 14) | 0.7 |
| | VF or FF | 44/186 | 23.7% | | 3.7 (2.8-5.2; n=186; e=145) | | -3.1 (-37.5 to 11.3, n = 183) | |
| **FCGR3A/2A** | Favorable | 15/59 | 25.4% | 0.76 | 3.7 (1.96.2; n=59; e=47) | 0.72 | -4.8 (-57.7 to 8.9, n = 58) | 0.96 |
| | Unfavorable | 33/141 | 23.4% | | 3.9 (2.5-5.4; n=141; e=109) | | -2.8 (-37.5 to 11.9, n = 139) | |
| **FCGR3A/2C** | Favorable | 29/104 | 27.9% | 0.17 | 3.8 (2.8-5.5; n=101; e=83) | 0.88 | -6.4 (-55.1 to 7.3, n = 103) | 0.25 |
| | Unfavorable | 19/96 | 19.8% | | 3.7 (2.1-5.6; n=96; e=73) | | -0.5 (-32.4 to 12.8, n = 94) | |
| **FCGR3A/2A/2C** | Favorable | 15/59 | 25.4% | 0.76 | 3.7 (1.9-6.2; n=59; e=47) | 0.72 | -4.8 (-57.7 to 8.9, n = 58) | 0.96 |
| | Unfavorable | 33/141 | 23.4% | | 3.9 (2.5-5.4; n=141; e=109) | | -2.8 (-37.5 to 11.9, n = 139) | |

**Table S7: FCγR results for IO-naïve patients.** Associations of FCγR genotypes with the three clinical outcome parameters (OR, PFS, and tumor shrinkage) within the IO-naïve patients. The definitions for the FCγR genotype categories are detailed in Supplemental Table S6. Abbreviation: CI, confidence interval; IQR, interquartile range.

## Supplementary Figure S1

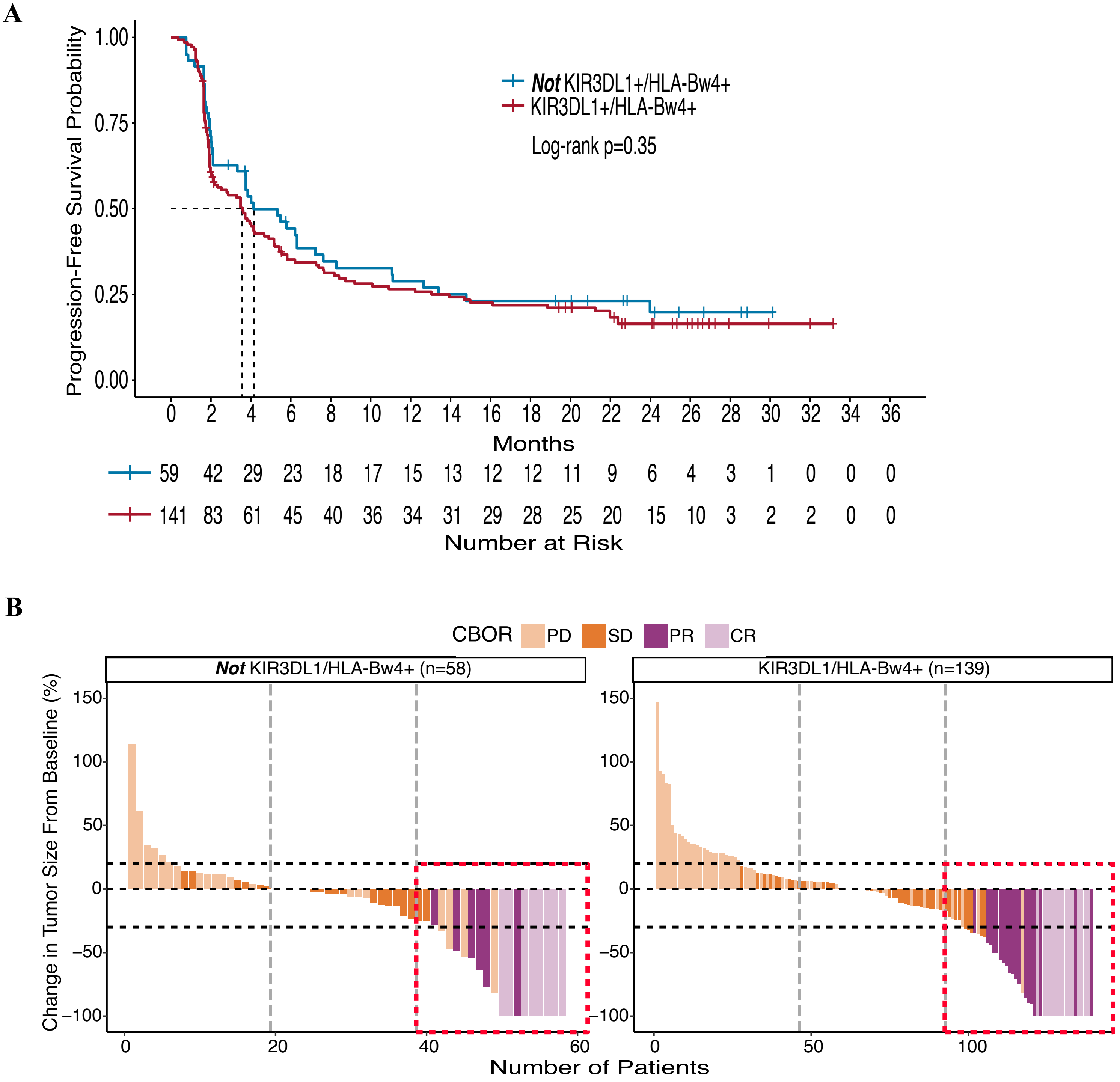


**Figure S1: KIR3DL1 and its HLA-Bw4 ligand does not influence clinical outcome.** Waterfall plots displaying OR and tumor shrinkage (**a**) compares patients who are KIR3DL1+/HLA-Bw4+ (right) with patients who are ***not*** KIR3DL1+/HLA-Bw4+ (left). CBOR is the confirmed best overall response by RECIST 1.1 criteria; PD, progressive disease (light orange); SD, stable disease (orange); PR, partial response (purple); CR, complete response (light purple). Vertical dotted lines divide the number of patients into thirds, and horizontal dotted lines indicate a +20% increase and -30% decrease in target lesion size from baseline. Dotted red box outlines the top third percent of patients with a positive clinical response. Kaplan-Meier curve for PFS **(b)** compares patients who are KIR3DL1+/HLA-Bw4+ (red line) to patients who are ***not*** KIR3DL1+/HLA-Bw4+ (blue line).
